# Association between tea consumption and gastroesophageal reflux disease: A meta-analysis

**DOI:** 10.1097/MD.0000000000014173

**Published:** 2019-01-25

**Authors:** Hongying Cao, Xiaoyi Huang, Xiaosong Zhi, Cuihong Han, Liang Li, Yuyi Li

**Affiliations:** a Department of Pathology, Jining No.1 People's Hospital, Jining, Shandong; b Department of Pathology, Changhai Hospital; c Department of Cell Biology, Second Military Medical University, Shanghai; d Department of Proctology, Jining No.1 People's Hospital, Jining, Shandong, China.

**Keywords:** diet, gastroesophageal reflux disease, meta-analysis, tea

## Abstract

**Background:**

Gastroesophageal reflux disease (GERD) is one of the most common digestive system diseases, which is associated with lifestyle and dietary factors. The main mechanism involved in GERD is affected by demographics, lifestyles, and dietary factors. Tea consumption is reported to be associated with GERD, especially in Asian population. However, the effect of tea drinking on GERD risk is still controversial. The aim of this study was to investigate the relationship between tea consumption and the risk of GERD by meta-analysis.

**Methods:**

We searched the published research databases such as PubMed and Embase for studies that were published up to March 2018. The search results were reviewed by 2 authors, and studies that complied with the criteria were selected. Odds ratio (OR) and 95% confidence interval (CI) were used to assess the association between tea consumption and the risk of GERD.

**Results:**

Twenty-three articles including 30 studies were included in the meta-analysis. The result of meta-analysis showed that tea drinking had no significant association with the risk of GERD. The odds ratio (OR) and 95% CI were 1.12 and (0.98–1.27). In subgroup analysis based on geographical region, tea consumption can increase the risk of GERD in East Asia (OR = 1.27, 95% CI = 1.07–1.51), while the risk of GERD was decreased in Middle Asia (OR = 0.77, 95% CI = 0.63–0.95). Besides, in the subgroup of study design, there was a significant association between tea intake and the GERD in cross-sectional study. In no symptom subgroup, the risk of GERD was increased (OR = 1.47, 95% CI = 1.11–1.93).

**Conclusions:**

There was no significant relationship between tea consumption and the risk of GERD overall. However, in subgroup analysis, tea drinking may increase the risk of GERD in East Asia and decrease in Middle Asia. To clarify the causality between tea intake and GERD, a more precise study design will be needed.

## 1 Introduction

Gastroesophageal reflux disease (GERD) is diagnosed based on the clinician's symptom assessment such as acid regurgitation and heartburn. GERD is a very common gastrointestinal diagnosis and the incidence rate is on the rise. The prevalence of GERD in North America and Europe is 8.8% to 27.8%, while it is only 2.5% to 7.8% of the population in East Asia.^[1]^

The main mechanism involved in GERD is transient low sphincter relaxation episodes and decreased lower esophageal sphincter (LES) pressures.^[2]^ Such mechanisms of GERD are affected by demographics, lifestyles, and dietary factors. Among these factors, smoking and obesity are considered as the recognized factors.^[3,4]^ However, the relationship between GERD and other environmental factors is not yet clarified. Dietary factors such as fat and chocolate are putative risks of GERD, but dietary interventions are not effective in treating GERD.

Tea, the beverage processed from *Camellia sinensis*, has been considered a healthy drinking with beneficial effects like anti-aging and antidiabetic for thousands of years.^[5]^ However, tea is also related to some clinical symptoms including heartburn and reflux.^[6,7]^ Tea consumption is reported to be associated with GERD, especially in Asian population. Theoretically, theophylline, a component of tea, may contribute to relax the LES, leading to esophageal acid reflux.^[8]^ In observational studies, some case–control studies have reported that there is no correlation between GERD and tea consumption,^[9,10]^ while other studies suggested a positive association between tea drinking and the risk of GERD.^[11–13]^ Thus, the effect of tea drinking on GERD risk is still controversial. The aim of this study is to perform a meta-analysis on the relationship between tea drinking and the incidence of GERD.

## 2 Materials and methods

### 2.1 Literature search

A literature search was performed based on PubMed and Embase databases for studies that reported the association between tea intake and the risk of GERD up to December 20th, 2017. The following keywords and their corresponding MeSH terms were used: “tea” or “lifestyle” or “diet” or “dietary” combined with “gastroesophageal reflux” or “GERD” or “esophageal reflux disease” or “esophagitis” or “Barrett's esophagus.”

### 2.2 Selection and exclusion criteria

The studies were included by the following selection criteria: a case–control or cohort study design or cross-sectional study; providing information related to association between tea intake and the risk of GERD; providing the odds ratio (OR) or relative risk (RR) data as well as the corresponding 95% confidence intervals (CI) for the highest versus the lowest level of tea intake, or necessary data for calculation.

The studies were excluded by the following exclusion criteria: nonepidemiologic studies (e.g., review, meta-analysis, case report, editorial, or human-uncorrelated experiment); duplicated study; not providing sufficient data to calculate OR (or RR) and CI. Other reasons (not pure tea such as tea with salt; tea with milk, tea and coffee combined) for which the studies were not appropriate to be involved in the meta-analysis.

### 2.3 Data extraction and quality assessment

The following key information was recorded: the last name of the first author, publication year, country, geographical region, study design, number of cases and controls or total sample size, exposure assessment, diagnosis method, disease type, OR (or RR), the corresponding 95% CI, and the covariates adjusted for in the analysis. Quality assessment was conducted according to the Newcastle–Ottawa scale (NOS). The NOS is divided into 3 parts: selection (4 points), comparability (2 points), and exposure/outcome assessment (3 points). The study with an NOS ≥ 6 was considered as high quality and included in our meta-analysis. The process of data extraction was carried out by 2 independent authors with a standardized form based on inclusion and exclusion criteria mentioned above. Any divergence was resolved by revaluation until consensus was reached. The meta-analysis was approved by the Hospital Ethics Committee (Jining No. 1 People's Hospital) to not re-identify the participants.

### 2.4 Statistical analysis

The OR and the 95% CI values were directly extracted from each study, or were calculated from the raw data. For studies where the study population was divided into groups based on the amount of tea intake, we used the groups with the highest and lowest levels of tea intake among the various categories. Pooled analysis was performed to evaluate the association between tea consumption and the risk of GERD. The pooled analysis was shown as forest plot. Chi-square-based *Q* statistic test via *I*^*2*^ value and *P* value was used to analyze the statistical heterogeneity, which depicts the percentage of variation across studies due to heterogeneity rather than chance.^[14]^ An *I*^*2*^ value of >50% was considered to indicate substantial heterogeneity. When the *I*^*2*^ value was >50%, the random effects model was used, and when it was <50%, the fixed effects model was used. Subgroup analyses according to study design, ethnicity, exposure assessment, and diagnosis method, was performed to assess the potentially important covariates that might exert substantial impact on between-study heterogeneity. Publication bias was analyzed by using Egger's test and Begg's funnel plot.^[15]^ Sensitivity analysis was conducted to describe how robust the pooled estimator was when removing an individual studies at a time. STATA version 12.0 (Stata Corp LP, College Station, Texas) was used for the whole meta-analysis. Statistical significance was set at *P < *.05.

## 3 Results

### 3.1 Study selection and characteristics

**Figure 1:**
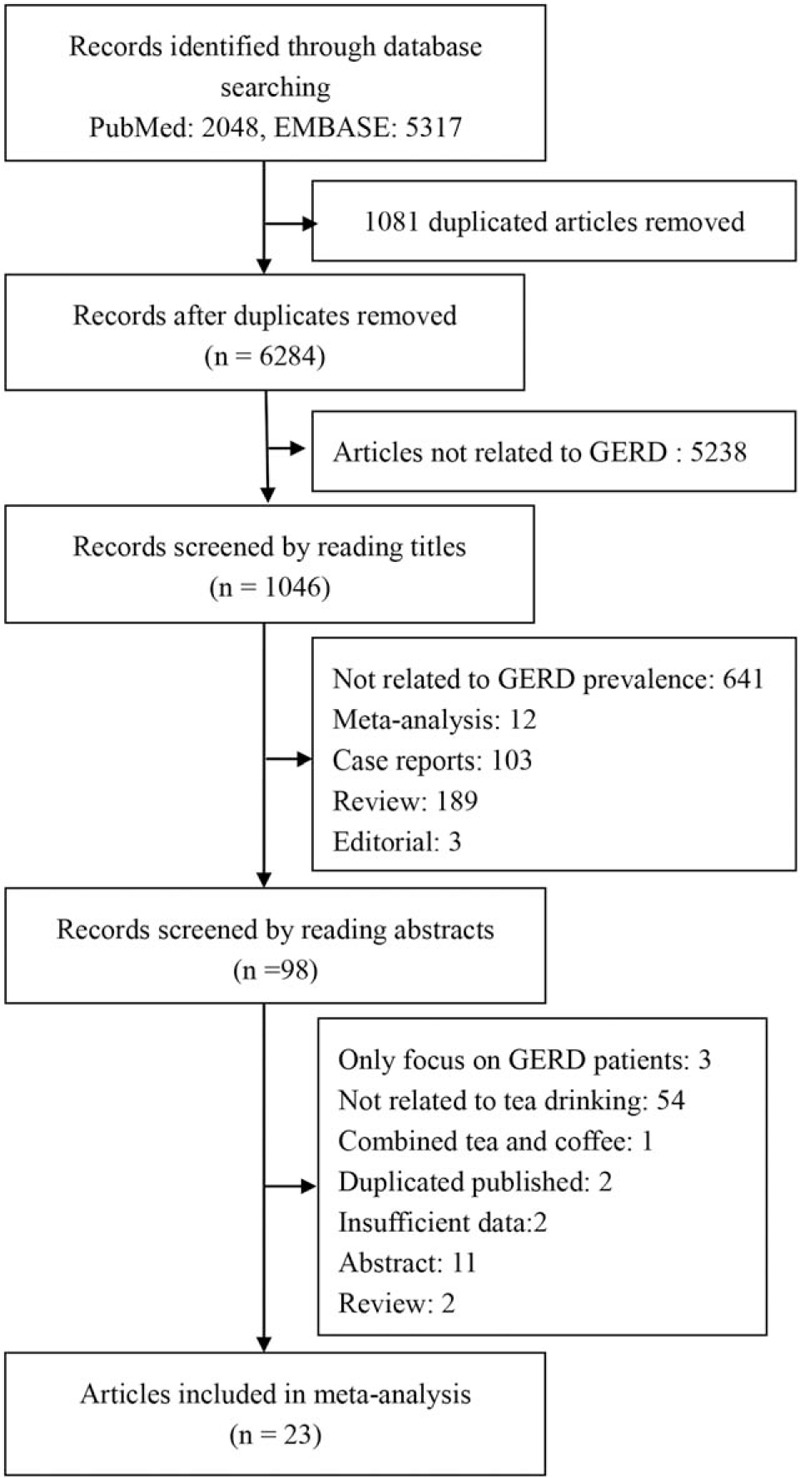
The flow diagram of study selection and inclusion process.

The detailed study screening processes were shown in Figure 1. Database search led to retrieval of 2048 records from the database of PubMed, and 5317 records from Embase, among which there were 1081 duplicated records. Then after reviewing the titles and the abstracts, 98 studies were identified. Then we reviewed the full article, and found 23 eligible studies, which were included in the final subject studies for meta-analysis (Fig. 1).

The characteristics of the included studies on tea intake and the risk of GERD are presented in Table 1. The studies were published from 2004 to 2017. Since the subjects could be divided into different groups according to the disease types (e.g., gastroesophageal reflux disease, GERD; nonerosive gastrooesophageal reflux disease, NERD; reflux esophagitis, RE; erosive esophagitis, EE; Barrett's esophagus, BE), studies by Murao et al, Du et al, Nam et al, and Filiberti et al were divided into 4, 3, 2 and 2 studies, respectively.^[11,16–18]^ Therefore, there were 30 estimates of 23 articles in our meta-analysis, among which, 17 were performed in East Asia, 5 were in Europe, 5 were in Middle East, 2 were in North America, and 1 was in South Asia. According to study design, 18 were cross-sectional studies, 9 were case–control studies, and 3 were cohort studies. Among them, 21 were conducted by questionnaire, 9 by interview. For disease diagnosis method, subjects in 13 studies were confirmed by symptom only and those in the other 17 studies by endoscopy.

**Table 1:**
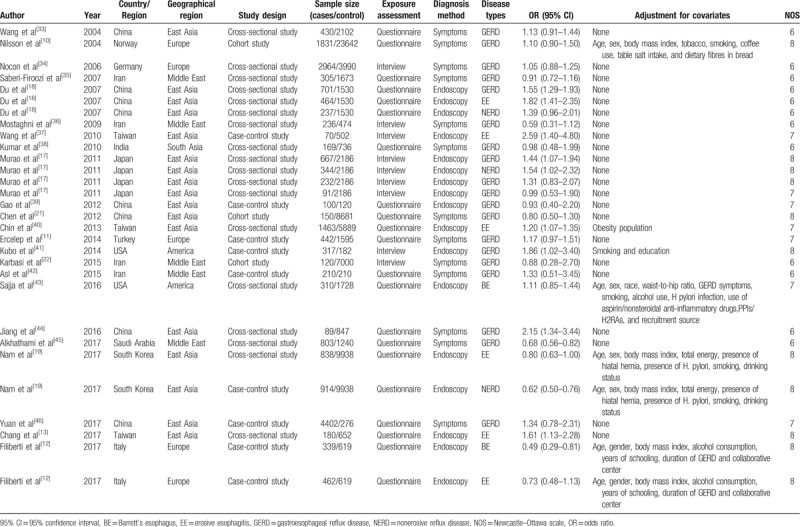
Characteristics of studies selected for the meta-analysis.

### 3.2 Tea consumption and risk of GERD

**Figure 2:**
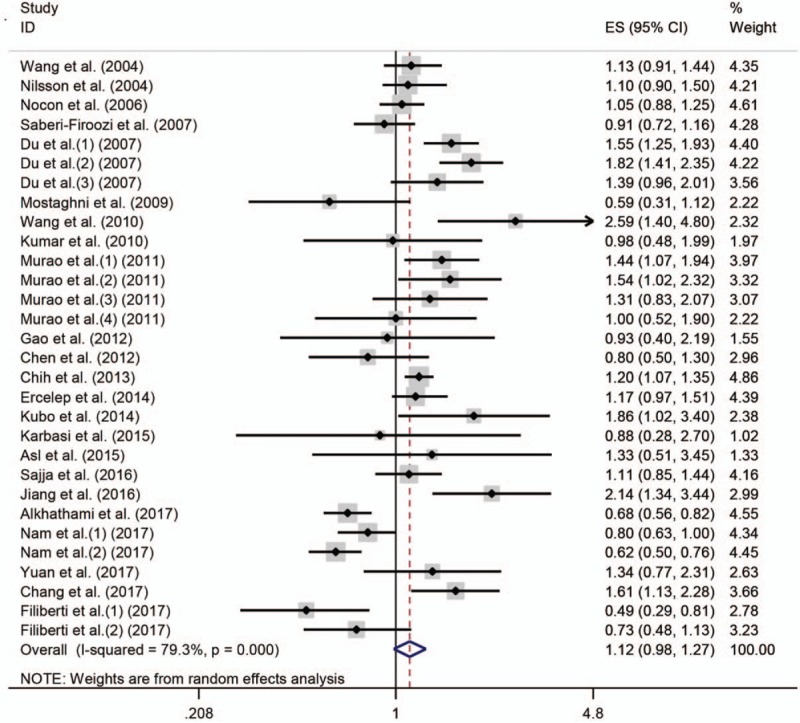
Forest plot for assessment of association between tea consumption and GERD risk in random effect model.

Generally, there was no significant correlation between the tea intake and GERD in our meta-analysis (OR 1.12, 95% CI 0.98–1.27), and these studies showed a significant heterogeneity (*I*^*2*^ = 79.3%, *P < *.001) (Fig. 2)

**Figure 3:**
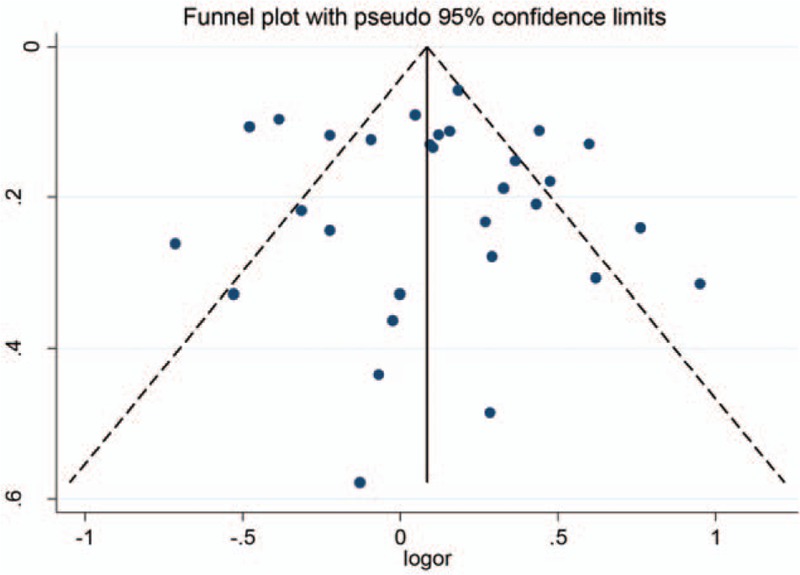
Funnel plot for assessment of publication bias.

**Figure 4:**
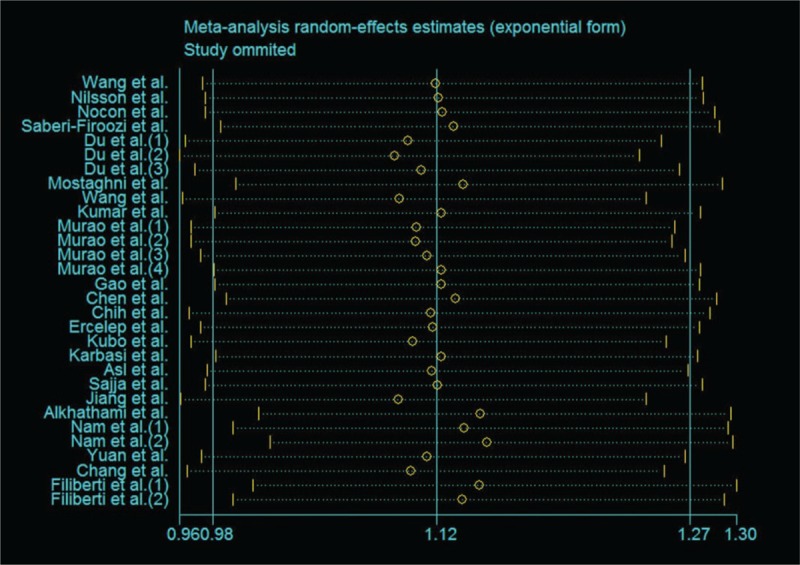
Sensitivity analyses for assessment of association between tea consumption and the risk of GERD. GERD = gastroesophageal reflux disease.

Whether the result of the research has publication bias or not was showed in Figure 3. It showed that all the studies were in a symmetrical distribution. The Egger's test (*P = *.674) also showed that there is no publication bias of the meta-analysis about tea intake and GERD. The sensitivity analysis was conducted to assess the stability of the result. When each article was omitted successively, the corresponding result did not change significantly (Fig. 4), which indicated that the validity were guaranteed.

### 3.3 Meta regression and subgroup analysis

We conducted the meta-regression to explore the observed heterogeneity of the meta-analysis. The result was shown in Table 2. In the meta-regression, the geographical region and symptoms contributed about 25% and 15% heterogeneity, respectively. In order to explore the significant between-study heterogeneity in the overall analysis, the studies were stratified by geographical region, study design, exposure assessment, and diagnosis method, the subgroup analysis results were showed in Table 3. In East Asia, tea intake was an increasing risk for GERD (OR 1.27, 95% CI 1.07–1.51), while in Middle East (OR 0.78, 95% CI 0.63–0.93), the risk was decreased. For the study design, cross-sectional studies suggested that tea intake increased the risk of GERD (OR 1.18, 95% CI 1.02–1.36); however, case–control study (OR 1.04, 95% CI 0.74–1.46) and cohort study (OR 1.02, 95% CI 0.82–1.27) showed no significant results. For exposure assessment, studies conducted by interview indicated that tea was a risk factor of GERD (OR 1.29, 95% CI 1.02–1.62), whereas studies carried out by questionnaire showed no significant correlation (OR 1.07, 95% CI 0.91–1.24). In addition, studies in which subjects were either diagnosed by symptom only (OR 1.02, 95% CI 0.87–1.20) or endoscopy (OR 1.19, 95% CI 0.99–1.43) did not show significant association between tea intake and GERD.

**Table 2:**
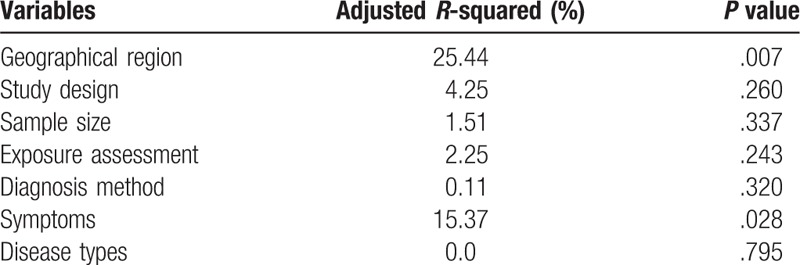
The result of meta-regression of the meta-analysis.

**Table 3:**
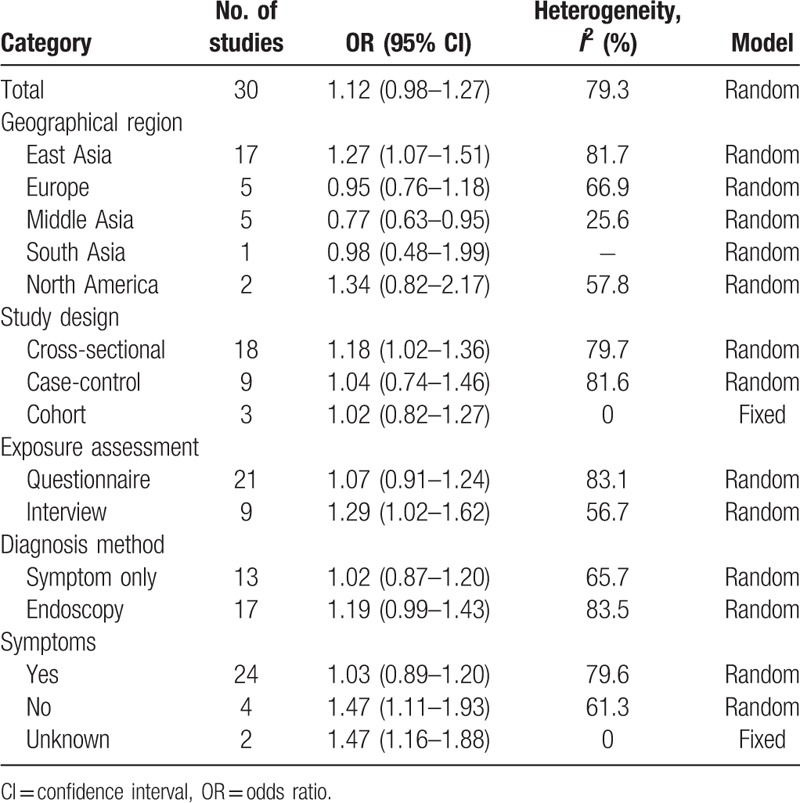
Subgroup analyses for tea consumption and risk of GERD by categories.

## 4 Discussion

In recent years, studies have been performed to assess the association between dietary factors and the risk of GERD. Some dietary factors like alcohol are proved to increase GERD risk. However, other dietary factors including tea drinking remain conflicting. To the best of our knowledge, this is the first meta-analysis that focused on tea consumption and the risk of GERD. Our meta-analysis showed that there was no significant association between tea consumption and the risk of GERD. Subgroup analyses based on exposure assessment and diagnostic method also failed to show a significant relationship between tea intake and the risk of GERD. However, increase risk was showed in Asian population with high tea intake based on ethnicity. Besides, we found that the risk of GERD increased in cross-sectional study of study design subgroup.

Previous studies conducted in Chinese population showed that strong tea drinking might association with the risk of GERD.^[20]^ The LES pressure is one of the motor mechanisms to prevent gastroesophageal reflux physiologically. In a randomized double-blind study, Gudjonsson and colleagues^[7]^ reported theophylline reduced the LES pressure and increased gastroesophageal reflux in normal adults.

Many epidemiological studies suggested that there is no significant relationship between tea drinking and GERD. However, some recent studies reported that frequent tea drinking might association with the risk of GERD. Chang et al^[12]^ conducted a cross-sectional study to evaluate the effect of regular tea drinking on asymptomatic erosive esophagitis. They found that the risk of asymptomatic erosive esophagitis was greater in those drinking tea than nondrinkers.^[13]^ Another study performed by Filiberti et al^[12]^ suggested that tea consumption could be protective factor for Barrett's esophagus or esophagitis. However, this is a case–control study based on limited subjects. To eliminate the causality between GERD and tea, more comprehensive study design or large scale prospective study will be needed.

Among the 30 studies of 23 articles including in this meta-analysis, 17 were performed in East Asia, 5 were in Europe, 5 were in Middle East, 2 were in North America, and 1 was in South Asia. In subgroup analysis by geographical region, there was a significant association between tea and the risk of GERD in East Asia. Tea is more popular in Asian countries than Western countries, and the amount of tea drinking are larger in Asian countries, such as China and Japan.^[9]^ This may lead to prominent result in East Asia. As for study design, most of the included studies are cross-sectional study, and only 3 studies are cohort study. As is known, cross-sectional study is a descriptive study, which has many disadvantages, such as nonresponse bias. Thus, cross-sectional study cannot reveal causality because of its weaknesses of aggregated data. Cohort study is a prospective study design, which may suggest the association between tea and GERD is not a direct causality. The number of studies using cohort study design are only 3,^[10,21,22]^ including 2 on Caucasian and 1 on Asian. To clarify the relationship between tea and the risk of GERD, more cohort study is needed to conduct in Asian population, especially in those who prefer strong tea. There are 2 main methods to diagnose GERD in these studies, namely the Gastroesophageal Reflux Questionnaire (GERQ) and endoscopy assessment.^[23,24]^ In clinical practice, GERD can be determined by common symptoms, such heartburn and acid reflux alone. However, this may be a confounding factor to evaluate the relationship between tea and GERD, since theophylline containing in tea can release chest pain.^[25]^ Besides, content of questionnaire may be another contributor of the heterogeneity in meta-analysis.

Besides tea drinking, other dietary and lifestyle factors such as coffee intake, alcohol drinking, smoking and obesity may related to the risk of GERD.^[4,26]^ There are many studies focused on the risk of GERD and coffee drinking.^[27,28]^ However, Kim et al^[29]^ conducted a meta-analysis to evaluate the association between coffee drinking and gastroesophageal reflux disease and found no significant association between the risk of GERD and coffee drinking. This meta-analysis revealed that coffee cannot increase the risk of GERD, even though some study suggested that coffee might change the lower esophageal sphincter pressure.^[30]^ Alcohol is another potential risk factor related to GERD. Recently, a meta-analysis revealed that alcohol intake might increase the risk of GERD.^[31]^ This finding suggested that drinkers should consider to limit the alcohol consumption to prevent the potential injury to the esophagus. Similar to coffee and alcohol, tea is a popular beverage worldwide. Our meta-analysis revealed that tea consumption was not associated with GERD worldwide. But in some geographical region, tea might be a potential risk factor of GERD, such as East Asia and Middle Asia.

To explore the observed heterogeneity, we conducted meta-regression to evaluate the study factors such as geographical region, study design, sample size, exposure assessment, diagnosis method, symptoms, and disease types. The result revealed that the adjusted *R*-squared in geographical region and symptoms were 25.44% and 15.37%, respectively. Thus, we conducted subgroup stratified by geographical region, symptoms, study design, exposure assessment, and diagnosis method. In this way, we can reduce the impact of high heterogeneity on the quality of the study to a certain extent. There are some limitations in the present meta-analysis. Firstly, the amount of tea consumption was quite heterogeneous among these studies. We used the highest and lowest exposure of tea intake in each study, though the amount of each study is not the same. In addition, the type of tea, degree of fermentation, the concentration of tea polyphenols and the temperature of tea are also confounding variables in meta-analysis. Secondly, some studies are based on the mixed ethnicity, which may ignore the gene–environment interaction on the risk of Barrett's esophagus.^[32]^ Thirdly, most of studies were cross-sectional study and case-control study. They cannot indicate the direct causality between tea drinking and the risk of GERD. Besides, the majority of the studies did not determine adjustment for confounding variables during the investigation. Fourthly, there exist many types of tea, such as green tea, oolong tea and black tea, whose degree of fermentation, the concentration of tea polyphenols and the temperature of the tea are confounding variables in our meta-analysis. Due to the limited data provided by the previous studies, we cannot clarify these potential heterogeneities in our meta-analysis. Last but not least, we excluded the study focused on the tea with salt, tea with milk, tea, and coffee combined, since these ingredients may have impact on GERD. These limitations can be a cause of heterogeneity in each study, which may affect the outcome of this meta-analysis.

In conclusion, there was no significant relationship between tea consumption and the risk of GERD overall. However, in subgroup analysis, tea drinking may increase the risk of GERD in East Asia. This suggested that tea drinking may be a potential risk of GERD and should be consider to limit the amount of intake in some GERD patients. Better-designed study is needed to confirm the effect of tea on GERD.

## Author contributions

**Formal analysis:** Yuyi Li.

**Software:** Xiaosong Zhi, Cuihong Han.

**Supervision:** Liang Li, Yuyi Li.

**Writing – original draft:** Hongying Cao, Xiaoyi Huang.

**Writing – review & editing:** Xiaosong Zhi, Liang Li, Yuyi Li.
