# Association between tea consumption and gastroesophageal reflux disease: A meta-analysis: Erratum

**DOI:** 10.1097/MD.0000000000014915

**Published:** 2019-03-08

In the article, “Association between tea consumption and gastroesophageal reflux disease: A meta-analysis”,^[1]^ which appeared in Volume 98, Issue 4 of *Medicine*, the journal name in reference 3 is incorrect and the reference should be:

[3] Eslami O, ShahrakiM, Bahari A, et al. Dietary habits and obesity indices in patients with gastro-esophageal reflux disease: a comparative crosssectional study. BMC Gastroenterol 2017;17:132.
